# Upper and lower respiratory airway complaints among female veterinary staff

**DOI:** 10.1007/s00420-021-01798-5

**Published:** 2021-10-20

**Authors:** F. Hoffmeyer, A. Beine, A. Lotz, O. Kleinmüller, C. Nöllenheidt, E. Zahradnik, A. Nienhaus, M. Raulf

**Affiliations:** 1 Institute for Prevention and Occupational Medicine of the German Social Accident Insurance, Institute of the Ruhr-Universität Bochum (IPA), Bürkle-de-la-Camp-Platz 1, 44789 Bochum, Germany; 2 Institute for Health Service Research in Dermatology and Nursing (IVDP), Center for Epidemiology and Heath Service Research in Nursing (CVcare), Universitätsklinikum Hamburg-Eppendorf (UKE), Hamburg, Germany; 3 Department of Occupational Medicine, Hazardous Substances and Health Research (AGG), Institution for Statutory Accident Insurance and Prevention in the Health and Welfare Services (BGW), Hamburg, Germany

**Keywords:** Asthma, Atopy, Occupational health, Rhinitis, Risk factor, Sensitization, Veterinary staff

## Abstract

**Objective:**

Working with animals is characterized by exposure to particulate, biological or chemical matter, and respiratory complaints are common. The aim of our cross-sectional study was to assess the prevalence of respiratory symptoms and diagnoses among veterinary staff.

**Methods:**

Participants working in veterinary practices were examined and a detailed questionnaire was used to collect data. IgE tests to common and animal allergens were performed to specify sensitization. Associations with respiratory outcomes were analysed using logistic regression models while controlling for potential confounders.

**Results:**

Atopy was seen in 31% of the 109 female participants. Symptoms of rhinoconjunctivitis were the most frequent complaints (*n* = 92; 84%). In 18% the diagnosis was confirmed by physicians. Symptoms of upper and lower airways were highly correlated and an asthma diagnosis was confirmed in 11% of participants. Modelling revealed that sensitization against cats/dogs was a significant risk factor for respiratory symptoms of upper [odds ratio (OR) 4.61; 95% confidence interval (CI) 1.13–18.81] and lower airways (OR 5.14; 95% CI 1.25–21.13), physician-confirmed rhinoconjunctivitis (OR 13.43; 95% CI 1.69–106.5) and asthma (OR 9.02; 95% CI 1.16–70.39) in assistant staff of small-animal practices.

**Conclusions:**

In several cases, rhinoconjunctivitis worsened after entering the profession. Atopy and specific sensitization to cats/dogs were risk factors for health impairments. Thus, to implement preventive measures, veterinary practice staff should be educated that upper respiratory tract symptoms are not harmless and should be diagnosed and treated early.

## Introduction

Veterinary medicine workers have an increased risk of sensitization, development of allergies, and occupational respiratory disease as a result of the field’s many and varied sources of exposure (Lutsky et al. 1985; Samadi et al. 2013). Respiratory diseases can be classified as allergic and non-allergic based on inflammatory mechanisms. Allergic rhinitis and allergic asthma are two well-known allergic respiratory diseases that may occur due to handling animals (Elbers et al. 1996). In rather rarer cases, hypersensitivity pneumonitis (HP) may be induced through inhalation of fungi- or animal-derived compounds (Kozak et al. 2012). On the other hand, exposure to irritative or toxic agents may result in non-allergic rhinitis, non-allergic asthma, bronchitis, and chronic obstructive pulmonary disease (COPD) (Tielen et al. 1996; Guerra 2005).

In Germany, 1016 occupational diseases were reported among employees in veterinary practices between 2007 and 2011, with occupational asthma induced by allergens ranked as the second most common occupational disease (28.8%) (Kozak et al. 2012). Veterinary assistants, like veterinarians, should be particularly considered when investigating allergen-induced occupational asthma (Nienhaus et al. 2005).

Studies have reported that among veterinarians the prevalence of symptoms ranges from 11% to 46% for dermal symptoms and from 40% to 60% for respiratory symptoms (Samadi et al. 2013). Specifically those working in large-animal practices or with farm animals and horses were reported to have an elevated risk of developing respiratory symptoms (Tielen et al. 1996; Samadi et al. 2013). In addition to the specific exposure conditions on site, personal characteristics, such as an atopic status and their private environment were also considered influencing factors for the development of respiratory complaints (Boulay & Boulet 2003; Samadi et al. 2012a). Overall, the current data on sensitization rates with regard to workplace-related or ubiquitous allergens and the prevalence of allergic symptoms of the upper and lower respiratory tract in veterinary medical staff are scarce or not available.

The main objective of this cross-sectional study conducted on veterinary staff was to evaluate self-reported respiratory symptoms and diseases, and serologically based sensitization. In addition, exposure assessment was included as part of the study design, and performed in the rooms of each practice, as well as the private homes of the participants, as described separately in detail (Zahradnik et al. 2021). Our examinations provide relevant information about upper and lower respiratory symptoms, which do not necessarily have to be associated with an allergic disease, taking into account the duration of the professional activity and the individual predisposition to atopy.

## Methods

### Participants and study design

The study was approved by a local Ethics Committee of the Ruhr University Bochum (registration number 17-6022) and was performed in accordance with the Declaration of Helsinki.

Information about the project and the first contact with the veterinary practices was primarily made by the Institution for Statutory Accident Insurance and Prevention in the Health and Welfare Services (BGW) and via announcements in various professional journals. Predominantly, veterinary practices located within a radius of about 50 km from Bochum (Germany) were included. Subsequently, practices within 75–100 km were contacted and subjects were recruited to reach the primary target number of 100 assistants. The owners of the practices were asked to inform their employees and motivate them to participate in the study and also to allow the exposure measurements in their practices. After obtaining the consent, the examinations were carried out on the voluntary study participants at the premises of the IPA. All study participants (*n*=122) gave written informed consent and received financial compensation for their participation.

### Questionnaire

A detailed questionnaire was used to collect data on the participant’s own and family history, allergic diseases in childhood and adolescence, as well as smoking habits and private animal contact. The overall time spent in veterinary medicine as a doctor or an assistant (profession years) and the employment in the practice under investigation (employment years) were assessed. Questions related to working conditions included the type of practice (small or large animals) and occupational animal contact in detail. In addition, during the examination current symptoms were recorded by a doctor’s interview. Questions concerning allergic rhinitis were taken from the European Community Respiratory Health Survey (ECRHS) and answers to those questions were used in different symptom scores as described in the following paragraph (Janson et al. 2001).

Rhinitis and conjunctivitis were defined by symptoms indicating irritation of nose or eyes (Hoffmeyer et al. 2014). Questions comprised runny nose, nasal congestion, itching nose, sneezing, and phlegm in the pharynx or watering eyes, burning and itching eyes. Answers to the questions were rated according to intensity of symptoms: 0 (no), 1 (weak), 2 (mild), and 3 (strong). The affirmative answers were integrated into additive symptom scores in the range of 0–15 (rhinitis, upper airways) and 0–6 (conjunctivitis). The sum of the conjunctivitis and rhinitis scores was termed rhinoconjunctivitis score (range 0–21). Symptoms of the lower airways were also rated according to intensity (0 to 3) and the additive score (up to 9) comprised the symptoms coughing or wheezing, excess phlegm or sputum and shortness of breath. Finally, the current prevalence with respect to a definitive doctor’s diagnosis was assessed for allergic rhinitis and asthma. Information was also obtained on the timing of the onset of respective symptoms in relation to the beginning of working as a veterinarian doctor or assistant. The relationship between work and respiratory symptoms was also addressed by asking whether symptoms have worsened at work in their cumulative occurrence or intensity.

### IgE determination

Measurements of total IgE and specific IgE to cat dander (e1), dog dander (e5), horse dander (e3), and the house dust mite (HDM) *Dermatophagoides pteronyssinus* (d1) were performed using ImmunoCAP 250 (ThermoFisherScientific, Uppsala, Sweden). Additionally, atopy status was determined with the inhalation allergy screening tool, sx1 (including *Dermatophagoides pteronyssinus*, cat and dog dander, timothy grass pollen, rye grass pollen, *Cladosporium herbarum*, birch pollen, mugwort pollen). Specific IgE values ≥ 0.35 kU/L were considered positive.

### Statistical analysis

Mean and standard deviation (SD) or median and interquartile range (IQR) were used to report measures of central tendency and dispersion of normally or non-normally distributed variables, respectively. Comparisons of stratified variables across two groups were done using *t* test or Mann–Whitney *U* test, as appropriate. The Kruskal–Wallis test was applied to compare more than two groups for a non-normally distributed variable.

Two-by-two contingency tables were analysed using Fisher’s exact test, bigger contingency tables were analysed with Pearson chi-squared test unless the expected cell counts of less than 5 comprised 25% or more of a table, in which case Fisher’s exact test was applied. Influences of profession and specific sensitization on respiratory symptoms and diseases were estimated. For a dependent dichotomous variable, like the presence of confirmed rhinoconjunctivitis and asthma, a logistic regression model (logit model) was used. To adjust for potential confounding, personal characteristics (age and smoking) known to affect respiratory symptoms and diseases were added to the model. We used a maximum likelihood approach and the iteratively reweighted least squares algorithm (Fisher scoring) to fit the different logistic regression models. For validation purposes, we used the Newton–Raphson algorithm instead of Fisher scoring, which led in all cases to very similar results (data not shown).

Symptom scores were categorized as follows and cumulative logit models were applied to predict the ordinal responses: rhinoconjunctivitis score, four categories “0”, “1–3”, “4–6”, and “≥7”; lower airway score, three categories “0”, “1–2”, and “≥3”. The association between lower and upper airway score values were analysed with Spearman’s rank correlation. The association between age and job duration is presented as squared Pearson correlation coefficient *r*^2^.

*p* values <0.05 were considered statistically significant, though for interpretation it should be noted that no correction for multiple comparisons was applied. The statistical analysis was performed with SAS, version 9.4 (SAS Institute, Inc. Cary, NC). The graphs were made with GraphPad Prism, version 8.4.1 (GraphPad Software, Inc., La Jolla, CA).

## Results

### Participating practices

Primarily, 174 eligible practices located within a radius of 50 km were contacted. Of these, 26 owners (15%) agreed to participate and 80 subjects could be motivated for the study. After expanding the radius, an additional 42 subjects from 19 practices were recruited. The recruitment was stopped after examination of 103 assistants (and 19 veterinarians).

### Subject characteristics

One hundred twenty-two participants in total were recruited for the study. Male participants were excluded due to their overall small number (*n* =12). Of the 12, 5 were veterinarians, 7 were assistants, and all but one worked in a practice treating only small animals. In addition, 1 female belonging to the assistant staff of a small-animal practice had to be excluded from further analyses due to an incomplete dataset. Characteristics, including serological parameters of the female participants with complete dataset (*n* = 109) stratified according to job title and practice speciality are shown in Table 1. No significant differences were observed concerning the anthropometric measures of the assistant staff working in small animal (group A), or mixed or large animal practices (group B). The duration of profession was similar in both groups with a median of 7 years. In contrast, the female veterinarians (group C) were significantly older (*p* < 0.0001), longer in profession (*p* = 0.004), and—though not significant—were less frequent current smokers (*p* = 0.121) compared to the assistant staff (groups A and B). Atopy, with respect to sx1 level, was identified in groups A, B, and C with a prevalence of 33.7%, 16.7%, and 28.6%, respectively. Sensitization to animals were most frequent for cats (*n* = 13; 11.9%) and dogs (*n* = 10; 9.2%) and comparable among all three subgroups. The majority of subjects were sensitized to both cats and dogs (*n* = 9; 8.3%) resulting in a sensitization against cats and/or dogs (cats/dogs) in 14 of 109 subjects analysed (12.8%). One subject demonstrated slightly elevated specific IgE levels to cat dander (0.44 kU/L) without being defined as atopic. Sensitization to horse allergens was detected in three subjects (2.8%), all of whom were co-sensitized to cat dander. Assistant staff and veterinarians working in small-animal practices showed enhanced sIgE levels to HDM in 13.3% (group A) and 14.3% (group C), respectively; whereas, no sensitization could be detected against HDM among the assistant staff working in other practices (group B). Veterinary medical staff were often pet owners (81.7%), e.g. dog 51.4%, cat 43.1%, and both 16.5%. In addition, 28.4% of the staff reported private contact with horses.

**Table 1:** Characteristics of the participating females stratified by job title and type of practice

	All; *n* = 109	Group A; *n* = 83	Group B; *n* = 12	Group C; *n* = 14	*p* value
Job title		Assistant staff	Assistant staff	Veterinarians	
Type of practice		Small animals	Mixed or large animals	Small animals	
Age (years) [median (IQR)]	31.0 (25.5; 43.0)	29.0 (25.0; 38.0)	27.5 (19.3; 40.8)	47.5 (42.3; 54.3)	< 0.0001
Smoking habits					
Current (*n*/%)	33/30.3%	29/34.9%	3/25.0%	1/7.1%	0.121
Former (*n*/%)	13/11.9%	11/13.3%	0	2/14.3%	
Never (*n*/%)	63/57.8%	43/51.8%	9/75.0%	11/78.6%	
Profession (years) [median (IQR)]	7.4 (3.3; 17.3)	6.8 (3.2; 15.2)	6.7 (2.4; 22.0)	18.3 (11.5; 24.8)	0.004
Employment (years) [median (IQR)]	4.0 (1.8; 11.3)	3.3 (1.8; 8.9)	6.7 (2.4; 22.0)	12.5 (5.2, 19.3)	0.017
sx1					
(*n*/%)	34/31.2%	28/33.7%	2/16.7%	4/28.6%	0.500
(kU/L)	3.64 (0.61; 14.76)	3.64 (0.64; 17.28)	1.56; 5.42	3.96 (0.43; 35.83)	
sIgE cat					
(*n*/%)	13/11.9%	10/12.1%	1/8.3%	2/14.3%	0.884
(kU/L)	1.66 (0.90; 12.16)	1.54 (0.96; 11.43)	2.13	0.44; 49.04	
sIgE dog					
(*n*/%)	10/9.2%	7/8.4%	1/8.3%	2/14.3%	0.737
(kU/L)	1.70 (0.56; 2.85)	1.95 (0.49; 2.76)	1.45	0.71; 3.12	
sIgE horse					
(*n*/%)	3/2.8%	1/1.2%	1/8.3%	1/7.1%	0.141
(kU/L)	1.05; 2.51; 4.44	2.51	1.05	4.44	
sIgE HDM					
(*n*/%)	13/11.9%	11/13.3%	0	2/14.3%	0.590
(kU/L)	1.37 (0.78; 12.87)	1.37 (0.97;12.24)		0.58; 17.42	

*p *value for group A vs group B vs group C, *IQR* interquartile range*, HDM* house dust mite (*Dermatophagoides pteronyssinus*)

sx1 and sIgE: specific IgE data are frequencies of values ≥ 0.35 kU/L, sIgE concentrations are presented as median with IQR, for up to *n* = 3 subjects individual results are given

### Symptoms and physician-confirmed diagnoses

Results on symptoms of the upper and lower respiratory tracts and physician-confirmed diagnoses are given in Table 2. The prevalence rates and frequencies for assistant staff in small-animal practices were 51.8% (*n* = 43) for conjunctivitis, 88.0% (*n* = 73) for rhinitis and 49.4% (*n* = 41) for lower airway symptoms. Assistant staff in the mixed or large-animal practices reported comparable levels of these symptoms, e.g. 41.7% (*n* = 5), 83.3% (*n* = 10), and 25.0% (*n* = 3), respectively. All subjects reporting signs of conjunctivitis also complained about rhinitis. Thus, the number of people with rhinoconjunctivitis (rhinitis and/or conjunctivitis) symptoms corresponded to the number of people with rhinitis; in minor cases subjects primarily complained about affected eyes. Among the small-animal practice staff, 20.8% (*n* = 16) of assistants (Group A) and 14.3% (*n* = 2) of veterinarians (Group C) were officially diagnosed with rhinoconjunctivitis by a physician. Asthma was diagnosed in 13.0% (*n* = 10) and 7.7% (*n* = 1) in both groups, respectively. Assistant staff of mixed or large animal practices (Group B) reported no physician-confirmed diagnosis of rhinoconjunctivitis or asthma.

**Table 2:** Allergic and respiratory symptoms and physician-confirmed diagnoses stratified by job title and type of practice

	All; *n* = 109	Group A; *n* = 83	Group B; *n* = 12	Group C; *n* = 14	*p* value
Job title		Assistant staff	Assistant staff	Veterinarians	
Type of practice		Small animals	Mixed or large animals	Small animals	
Conjunct. sympt. (*n*/%)	53/48.6%	43/51.8%	5/41.7%	5/35.7%	0.472
Rhinitis sympt. (*n*/%)	92/84.4%	73/88.0%	10/83.3%	9/64.3%	0.070
Rhinoconjunct. sympt. (*n*/%)	92/84.4%	73/88.0%	10/83.3%	9/64.3%	0.070
Lower AW sympt. (*n*/%)	52/47.7%	41/49.4%	3/25.0%	8/57.1%	0.215
RC diagnosis (*n*/%)	18/18.0%	16/20.8%	0	2/14.3%	0.349
Asthma diagnosis (*n*/%)	11/11.0%	10/13.0%	0	1/7.7%	0.736

*Conjunct.* conjunctivitis, *AW* airways, *RC* rhinoconjunctivitis

RC diagnosis: results reduced for group A (*n* = 77) and group B (*n* = 9) due to missing values

Asthma diagnosis: results reduced for group A (*n* = 77), group B (*n* = 10), and group C (*n* = 13) due to missing values

A high percentage of the respiratory diseases were work-related, e.g. aggravation of rhinoconjunctivitis was reported in 56.3% of the affected subjects in group A (*n* = 9) and all subjects in group C (*n* = 2); aggravation of asthma was work-related in 50% (*n* = 5) of the cases in group A. The incidence rates of new-onset respiratory diseases that could be attributed to work were 2.4% (*n* = 2) and 7.1% (*n* = 1) for rhinoconjunctivitis, and 6.0% (*n* = 5) and 7.1% (*n* = 1) for asthma in groups A and C, respectively.

### Respiratory symptoms and atopy

**Fig. 1:**
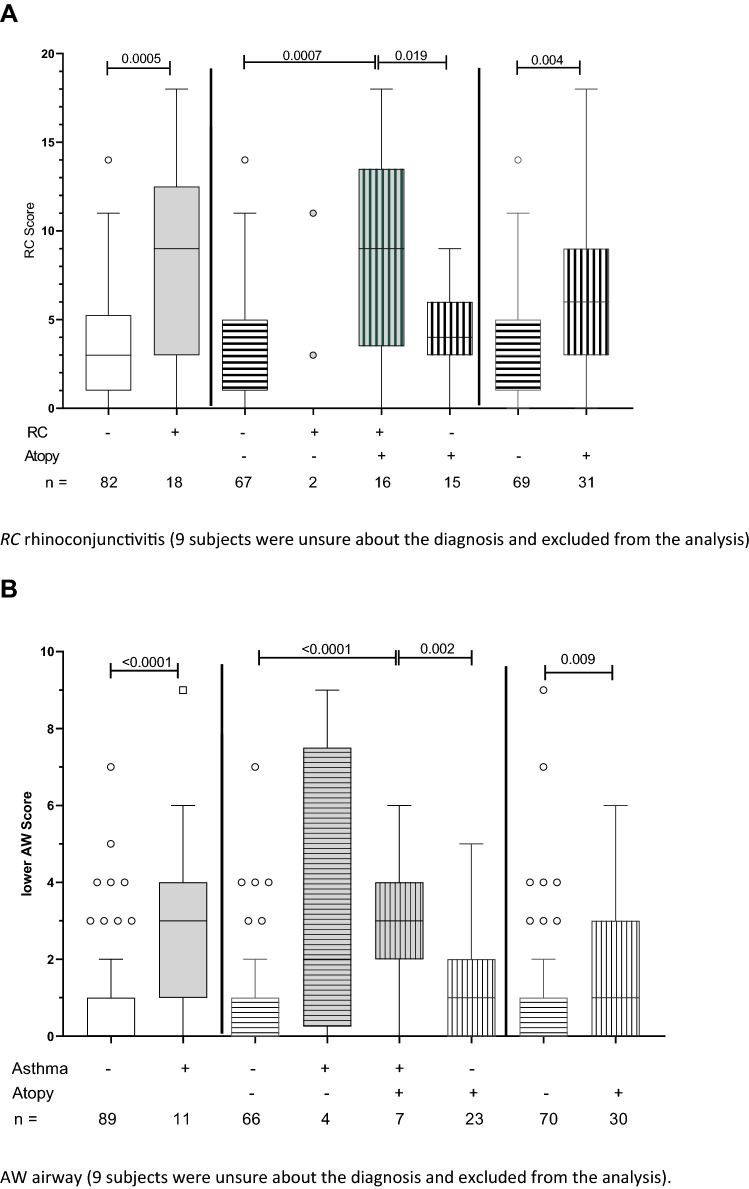
Symptom scores in employees stratified by physician-confirmed diagnosis and atopy [**A** rhinoconjunctivitis (RC) score and diagnosis, **B** lower airway score and asthma diagnosis]. *RC* rhinoconjunctivitis (nine subjects were unsure about the diagnosis and excluded from the analysis). AW airway (nine subjects were unsure about the diagnosis and excluded from the analysis). Boxes comprise the 25th and 75th percentiles and the median values, vertical bars show the 5th and 95th percentiles, and circles represent values outside of the given percentiles. Only *p* values for statistically significant differences are presented above the box plots, based on Mann–Whitney *U* test. Physician-confirmed diagnosis: *no* white bars; *yes* filled bars, atopy: *no* horizontal hatching; *yes* vertical hatching

The results described above demonstrate a high prevalence of eye and even higher number of nose complaints, suggesting underlying rhinoconjunctivitis. To verify this, we compared these complaints (rhinoconjunctivitis score) with the doctor’s diagnosis of rhinoconjunctivitis. Since irritative and allergic influences can be the causative factors of this complex of complaints, we tested the hypothesis that atopy influences the severity of respiratory symptoms. Figure 1A shows the severity of symptoms depending on the presence of atopy in subjects with physician-confirmed rhinoconjunctivitis among the veterinary staff. Subjects with an official rhinoconjunctivitis diagnosis had significantly more symptoms (stratified by RC score) than those without (*p* = 0.0005). The majority of subjects with rhinoconjunctivitis were also classified as atopics (16 of 18; 88.9%), and atopics had a significantly higher RC score than non-atopics (*p* = 0.004). In the absence of a disease diagnosis, fewer symptoms were reported, which showed no significant differences in relation to atopy status (*p* = 0.246).

**Fig. 2:**
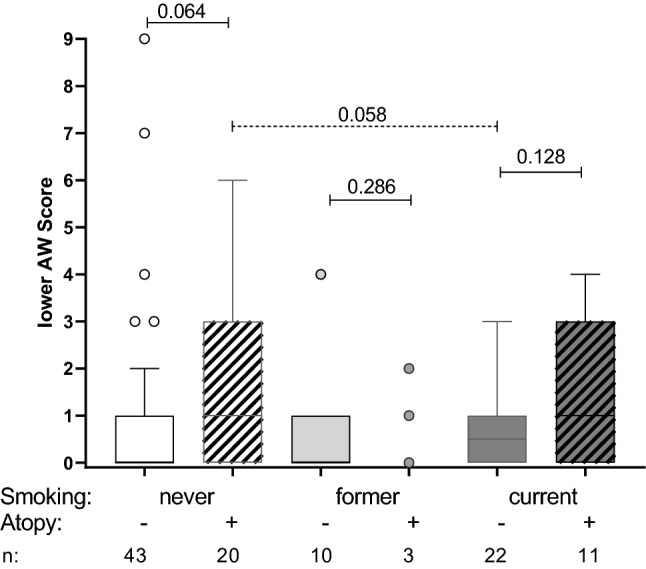
Symptoms of lower airways (AW score) in female veterinary staff stratified by smoking habits and atopic status. Boxes comprise the 25th and 75th percentiles and the median values, vertical bars show the 5th and 95th percentiles, and circles represent values outside of the given percentiles. *p* values for comparisons are presented above the box plots based on Mann–Whitney *U* test. Smoking: *never* white bars; *former* light gray bars, and *current* dark gray bars. Atopy: *yes* diagonal hatching

Accordingly, complaints of the lower airways were analysed with respect to underlying asthma (physician-confirmed diagnosis) and atopy (Fig. 1B). Again, those with physician-confirmed disease reported significantly more symptoms (*p* < 0.0001), which was independent of a positive or negative atopic classification. Although only eleven participants in total suffered from asthma, more than a third were non-atopics. Regardless of atopy status, however, there was a significant correlation between the upper and lower respiratory symptoms reported (Spearman correlation *r*=0.573; *p* < 0.0001; data not shown). Lower airway symptoms like cough, phlegm, and shortness of breath are also typical for chronic bronchitis or COPD resulting from smoking. Interestingly, the intensity of lower airway symptoms did not differ between never, former, and current smoker (Kruskal–Wallis test, *p* = 0.582). After additionally stratifying for atopy status, elevated lower airway scores were observed in atopics regardless of smoking habits. In particular, respiratory tract symptoms were reported more frequently by the never smoking but atopic subjects compared to the smoking non-atopics (*p* = 0.058) (Fig. 2).

### Duration of profession and atopy

The results above indicated an increased incidence of respiratory complaints and manifest (diagnosed) upper and lower respiratory diseases in the presence of atopy (serological sensitization to common allergens, sx1). The median job duration for non-atopic and atopic subjects was 8.3 and 6.7 years, respectively (*p* = 0.195). With respect to the total group of participants included in this evaluation (*n* = 109), 57.8% of the subjects were employed for less than 10 years (non-atopic 35.8%/ atopic 22.0%) and 42.2% for more than 10 years (non-atopic 33.0%/ atopic 9.2%). Thus, it appears that individuals identified as atopic tended to have shorter periods of employment. There was also no real difference in age between non-atopic (32.0; IQR 26.0–45.0 years) and atopic participants (27.5; IQR 24.0–40.0 years) (*p* = 0.222). A significant correlation (*p* < 0.0001 each) was observed between age and job duration for all (*r*^2^=0.607), non-atopic (*r*^2^=0.627), and atopic subjects (*r*^2^=0.579; data not shown).

### Modelling of personal and occupational factors related to symptoms and diseases

To further explore the relationship between personal characteristics, respiratory symptoms and diseases, multiple logistic regression models were applied (Table 3). Instead of atopy in general, we used specific sensitization for modelling. As shown in Table 3A, higher age was associated with a significantly elevated odds ratio (OR) for symptoms of lower airways (OR 1.98; 95% CI 1.24–3.17), and confirmed rhinoconjunctivitis (OR 2.31; 95% CI 1.10–4.88). There was also a trend for an association with asthma (OR 1.85; 95% CI 0.91–3.77). For participants working between 5 and 10 years, an increased risk of rhinoconjunctivitis (OR 3.18; 95% CI 0.82–12.3) was suggested. A trend for higher risks of respiratory symptoms (upper airways OR 2.15; 95% CI 0.73–6.35, lower airways OR 3.08; 95% CI 0.98–9.64) and diseases (rhinoconjunctivitis OR 4.17; 95% CI 0.91–19.1, asthma OR 3.26; 95% CI 0.62–17.0) were observed among staff demonstrating a specific sensitization to cats/dogs. Furthermore, specific sensitization to HDM must also be addressed as a potential risk factor for a physician-confirmed rhinoconjunctivitis (OR 4.13; 95% CI 0.93–18.4) but not asthma diagnosis. Finally, as expected, smoking habits could influence the prevalence of lower airway symptoms for current smokers (OR 1.79; 95% CI 0.77–4.19).

**Table 3:** Logistic regression analyses* of associations between exposure and prevalence of respiratory symptoms and diseases by subject characteristics

Table 3A	Upper AW score, *n* = 109			Lower AW score, *n* = 109			Rhinoconjunctivitis, *n* = 100			Asthma, *n* = 109		
	OR	95% CI		OR	95% CI		OR	95% CI		OR	95% CI	
Age (per 10 years)	1.12	0.74	1.69	**1.98**	**1.24**	**3.17**	**2.31**	**1.10**	**4.88**	1.85	0.91	3.77
Profession												
< 5 years	1			1			1			1		
5–10 years	0.70	0.28	1.78	0.65	0.22	1.86	3.18	0.82	12.35	0.81	0.13	4.94
> 10 years	0.66	0.23	1.86	0.54	0.17	1.71	0.09	0.01	0.81	0.42	0.06	2.94
Sensitization cats/dogs												
No	1			1			1			1		
Yes	2.15	0.73	6.35	3.08	0.98	9.64	4.17	0.91	19.09	3.26	0.62	17.04
Sensitization HDM												
No	1			1			1			1		
Yes	1.75	0.56	5.45	2.45	0.74	8.10	4.13	0.93	18.40	1.17	0.18	7.38
Smoking												
Never	–			1			–			–		
Former	–	–	–	0.66	0.18	2.42	–	–	–	–	–	–
Current	–	–	–	1.79	0.77	4.19	–	–	–	–	–	–

Table 3B	Upper AW score, *n* = 83			Lower AW score, *n* = 83			Rhinoconjunctivitis, *n* = 77			Asthma, *n* = 83		
	OR	95% CI		OR	95% CI		OR	95% CI		OR	95% CI	
Age (per 10 years)	1.24	0.75	2.03	**1.96**	**1.12**	**3.43**	**2.87**	**1.20**	**6.87**	**2.39**	**1.05**	**5.46**
Sensitization cats/dogs												
No	1			1			1			1		
Yes	**4.61**	**1.13**	**18.81**	**5.14**	**1.25**	**21.13**	**13.43**	**1.69**	**106.5**	**9.02**	**1.16**	**70.39**

*Upper and lower airway (AW) score: *cumulative logit model*; rhinoconjunctivitis and asthma: *logit model,* statistically significant values are marked in bold

When modelling was restricted to assistant staff working in small-animal practices (group A, *n*=83, Table 3B), which represented a more uniformly exposed group, then higher age, and specific sensitization to cats/dogs turned out to be risk factors for a statistically significant elevated OR of symptoms of upper, and lower airways, rhinoconjunctivitis and asthma, respectively.

## Discussion

Among the female veterinary staff examined in our study, the prevalence of upper and lower respiratory tract symptoms was 84% and 48%, respectively. Atopy, assessed by specific IgE to ubiquitous environmental allergens, was found in 31% of all study participants. Although the participants in our study were older (median age 31 years), the results correspond well with a previous study on predominantly female veterinary students (Samadi et al. 2012a), where 25% of the participants were found to be sensitized to at least one common allergen (house dust mites, grass mixture, birch pollen, cat, and dog fur). The results of that study (Samadi et al. 2012a) also showed that among the complaints, “rhinitis” was the most common (59%). Although (allergic) rhinitis has typical symptoms, only 18 individuals in our study reported that they were officially diagnosed with rhinitis by a physician. In the vast majority of these cases (89%), the subjects were also atopic, which was consistent with the hypothesis of an underlying allergic cause, e.g. allergic rhinitis. This result matches an earlier study from the UK reporting that only 18% of subjects with suspected allergic rhinitis visit a general practitioner for diagnosis (Bauchau and Durham 2004). Perhaps because of the typical symptoms, people take matters into their own hands with respect to self-diagnosis and treatment. However, if allergic rhinitis remains uncontrolled and untreated, more severe secondary problems can develop, including allergic asthma (Boulay and Boulet 2003). In agreement, most of our study participants with lower airway symptoms also reported concurrent upper airway symptoms, the intensity of which was highly correlated.

Our female veterinary staff group had an overall prevalence of current physician-diagnosed asthma of 11%, which is similar to the figures reported among female California veterinarians (Susitaival et al. 2003). Among veterinary medicine students in the Netherlands (80.3% women), 7.3% were officially diagnosed with asthma by a doctor (Samadi et al. 2012a). In comparison, the prevalence of asthma in a German survey (DEGS1) among female subgroups between 18–29 and 30–39 years was 12.8% and 9.4%, respectively (Langen et al. 2013). Compared to rhinitis, only 63.6% of our participants reporting asthma were identified as atopic. Veterinarian staff is exposed to animal dander, which includes important high molecular weight allergens. However, they are also exposed to irritative microbial components, such as endotoxin and β-glucan, and irritative substances like cleaning agents and disinfectants for most of their working hours (Samadi et al. 2012b, 2013). Thus, veterinarians and their staff are not only exposed to animal-related allergens, but also to relatively high levels of non-allergenic components known to induce airway irritation (Iversen and Pedersen 1990). Therefore, apart from an underlying allergic mechanism, an irritative asthmatic disease must also be considered.

Reports suggested that respiratory symptoms among veterinarians might be influenced by specific working conditions. In this respect, a study on veterinarians in the Southern Netherlands showed that chronic cough and chronic phlegm production were distinctly higher in veterinarians working in large animal practices with swine, poultry, and feeder calves primarily living in confinement buildings, compared to veterinarians working in other types of practices (Tielen et al. 1996). Furthermore, veterinarians, specifically those working with farm animals and horses, were reported to have an elevated risk of developing respiratory symptoms (Samadi et al. 2013). Ronmark et al. (2003) found that sensitization to horse specific allergens was a significant risk factor for the development of rhinitis and asthma. With this in mind, we stratified our participants according to type of praxis, e.g. handling small or large animals. Complaints of upper and lower airways were frequently reported by the veterinary staff in our study but regardless of the practice specialty. Also an association between sensitization to horse specific allergens and respiratory diseases could not be verified in our study. This can be accounted for in part by the low rate of serologically proven sensitization to equine allergens and because more than 75% of the study participants worked in small animal practices and, therefore, had no professional contact with horses. Our questionnaire also inquired about incidence of physician-confirmed respiratory diseases in relation to the period since they began working. This resulted in incidences of 2.8% (n=3/109) for rhinoconjunctivitis and 4.6% (*n*=5/109) for asthma, which agrees with an incidence of 3.9% based on self-reported symptoms indicative of asthma (Samadi et al. 2012a). About half of the asthma sufferers in our study also reported increased symptoms as a result of their work in veterinary practices.

Veterinary medical staff often have had contact to animals before beginning their professional career and during their leisure time, especially with pets or recreational animals (Das et al. 1992). This is not unexpected considering their choice of career in the field of veterinary sciences. A study on allergy among veterinary students revealed that 97% of the study population had previous contact with cats and/or dogs (Samadi et al. 2012a). According to the questionnaire, a large fraction of the participants in our study (81.7%) stated that they had private contact with animals, especially dogs (51.4%), cats (43.1%), and horses (28.4%). Data collected in our study on allergen exposure at the workplace and at home have already been published separately (Zahradnik et al. 2021).

Specific IgE to allergens of cat, dog, and horse was detected in 11.9%, 9.2%, and 2.8% of the veterinary staff, respectively. In a study among veterinary medicine students, 4.2%, 1.3%, and 1.6% of all participants were sensitized to these animals (Samadi et al. 2012a). Due to our cross-sectional study design, we could not determine the incidence of new-onset sensitization or allergy to animal allergens. Sensitization may have already occurred during childhood (Dotterud et al. 1997; Sander et al. 2018). Pets are common, and animal allergens are found in places like day care centers, schools, and public transport (Sander et al. 2018; Zahradnik and Raulf 2017).

Our results of an overall 12.8% sensitization prevalence to animal allergens are comparable to the DEGS1 survey with respect to female subgroups of same age. Using the same allergens, an overall sensitization rate of 14.7% (18–29 years) and 12.5% (30–39 years) was previously reported (Haftenberger et al. 2013).

Modelling showed that sensitization to furry animals was a consistent and major risk factor for respiratory symptoms and physician-confirmed respiratory diagnoses. This became statistically significant when the analyses were restricted to the veterinary staff of small animal practices. Sensitization to HDM was a relevant risk factor for physician-confirmed rhinoconjunctivitis. In addition, other airborne allergens (e.g. grass pollen) must be considered when discussing risk factors for allergic diseases. Despite some positive associations, smoking status did not significantly influence individual prevalence of symptoms or disease. Furthermore, a report on disease association with occupational exposures revealed that the prevalence of physician-diagnosed asthma did not differ with respect to smoking habits (Abrahamsen et al. 2017). Our results suggest that the atopy status or more specifically, sensitization to cats/dogs, might have an even greater impact on respiratory health than smoking in younger employees. We adjusted the analyses for age, which reflects lifetime exposure, whether at work or privately. While respiratory health burden increased with age, this was not shown for the duration of occupational employment. Rather the duration of employment in exposed workplaces was shown to be positively associated with the severity of the symptoms of airway diseases (Vermeulen et al. 2002). Our results show that individuals identified as atopic tended to have shorter periods of employment indicating that there might be confounding by some “healthy worker effect” (Stayner et al. 2003).

Allergic diseases and diseases caused primarily by irritation should be differentiated. While the former are characterized by clear signs of disease (especially wheezing, shortness of breath) with a direct relationship to exposure, COPD-type diseases tend to present gradually, and the clinically manifested diseases often appear only after decades. Respiratory symptoms observed in bioaerosol-exposed workers are thought to be mainly based on non-allergic inflammatory reactions (Eduard et al. 2001). Often, an increased risk of COPD defined by lung function impairment was seen for 10 working years (Hoffmeyer et al. 2014; Schantora et al. 2015). Moreover, irritative components, e.g. endotoxin, were suspected to mediate the onset of respiratory symptoms when working with farm animals or in pig barns. In accord, an association between the onset of respiratory symptoms and duration of animal exposure was reported in studies by Tielen et al. (1996) and Samadi et al. (2012a). Although our study group had no occupational exposure to farm animals, our results also indicate a relatively high prevalence of lower respiratory tract symptoms and asthma in non-atopics (non-allergic irritant asthma?), suggesting that non-allergic mechanisms may be the causative factor. 

We would like to explain some of the limitations of the study in the following. Although the contents of the study were communicated in advance in appropriate profession-specific journals and all practices were contacted according to the directories of the medical associations, the response in the primary study area within a radius of 50 km was lower than expected at 15%. Extended recruitment was slow, possibly because the travel costs (and time commitment) were no longer compensated for distances of up to 100 km. It could be speculated that individuals with an interest in health and pre-existing health problems in particular participated. Furthermore, it was important to recruit participants who worked in practices where the collection of airborne dust samples for allergen measurement was also possible (results published in Zahradnik et al. 2021). This again required the consent of the practice owners. Therefore, it cannot be ruled out that practice owners being aware of poor working conditions or health problems among their staff did not further motivate their staff to participate.

Another limitation is the cross-sectional design, which did not allow any deductions about causal relationships between the study variables. This study was based on self-reports of symptoms and physician-confirmed respiratory diagnoses. The differentiation between allergic and irritant-toxic forms of asthma is difficult. The detection of specific IgE to ubiquitous allergens may be a first indication of an allergic form. But even the detection of specific sensitization does not necessarily allow the diagnosis of allergic asthma. Ultimately, the causal link can only be proven with a high degree of probability by a specific challenge test, which was not possible in the scope of this study. As mentioned above, we were able to measure animal allergen levels at some practices but based on this single observation no exposure-effect associations could be estimated. A further limitation of our study is that we were not able to assess the exact nature and intensity of exposure to disinfectants and other toxic-irritant agents. But it is reasonable to assume relevant exposure for the veterinarian staff. As this workforce is dominated by women, it was not possible to recruit a gender-balanced collective.

A strength of our study is that we focused on assistants, about which there is only limited data on sensitization rates with regard to animal allergens and the prevalence of respiratory symptoms and diseases. Moreover, associations with respiratory outcomes were analysed using logistic regression models, controlling for potential confounders

In summary, veterinary assistant staff and veterinarians reported a high prevalence of respiratory complaints in the questionnaire. A relevant proportion of diseases, e.g. rhinoconjunctivitis worsened after subjects began working in the field. Atopy and specific sensitization against furry animals were risk factors for health impairment. Therefore, staff of veterinary practices should be made aware that upper respiratory tract symptoms are not harmless, to begin implementing preventive measures.

## Data Availability

The datasets generated during and/or analysed during the current study are available from the corresponding author on reasonable request.
